# Caregivers’ Perspectives on Discussions of Medical Treatment Preferences for People Living With Dementia Are Associated With Their Dementia Health Literacy and the Caregiving Relationship

**DOI:** 10.1093/geront/gnaf033

**Published:** 2025-01-29

**Authors:** Yuchen Zhang, Susan Sereika, Jennifer Seaman, Corinne Pettigrew, Marilyn Albert, Jennifer Lingler

**Affiliations:** Department of Acute & Tertiary Care, School of Nursing, University of Pittsburgh, Pittsburgh, Pennsylvania, USA; Department of Health & Community Systems, School of Nursing, University of Pittsburgh, Pittsburgh, Pennsylvania, USA; Department of Acute & Tertiary Care, School of Nursing, University of Pittsburgh, Pittsburgh, Pennsylvania, USA; Department of Neurology, School of Medicine, Johns Hopkins University, Baltimore, Maryland, USA; Department of Neurology, School of Medicine, Johns Hopkins University, Baltimore, Maryland, USA; Department of Health & Community Systems, School of Nursing, University of Pittsburgh, Pittsburgh, Pennsylvania, USA

**Keywords:** Alzheimer’s disease and related dementias, Dementia care, Health literacy, Advance care planning

## Abstract

**Background and Objectives:**

People living with dementia experience progressive functional decline and increased dependence on caregivers. This study examined the influence of caregivers’ dementia health literacy on perceptions of medical care preferences and advance care planning (ACP) in people living with dementia.

**Research Design and Methods:**

This analysis used data from a cross-sectional survey, “Care Planning for Individuals with Dementia,” administered nationwide by Alzheimer’s Disease Centers. We conducted binary, ordinal, and multinomial logistic regression.

**Results:**

On average, surveyed caregivers (*n* = 431) were 78.3 years, had 16 years of education, and were mainly White (88.5%). Most lived with (76.8%) and were the designated healthcare proxy (95.1%), with high dementia knowledge scores (mean = 8.4/10). As caregivers’ dementia knowledge scores increased, they were 1.27 times more likely (*p* = .02) to endorse comfort care. Caregivers with greater knowledge about severe dementia were less likely to need further treatment preference-related discussions (knowing a lot: odds ratio [OR] = 0.17, *p* < .001; knowing some things: OR = 0.37, *p* = .006). Caregivers live apart from patients were 2.71 times more likely to know about such discussions (*p* < .001). Caregivers of people in earlier stages endorsed greater needs for further conversations with clinicians (no impairment and mild cognitive impairment [MCI]: OR = 7.38, *p* = .002; mild impairment: OR = 5.32, *p* = .005) and their care recipients (no impairment and MCI: OR = 5.24, *p* = .02).

**Discussion and Implications:**

These findings highlight the role of dementia-specific education in ACP discussions among people living with dementia, caregivers, and healthcare clinicians. These findings are important because evidence suggests that ACP may promote quality of life, reduce iatrogenic harm, minimize healthcare overutilization, and alleviate care-related burdens.

## Background

By 2050, there will be approximately 12.7 million older adults affected by symptoms of Alzheimer’s disease and related dementias (ADRD) in the United States (Alzheimer’s Association, 2022). ADRD lead to progressively worsening functional, cognitive, and communicative capacities, which result in greater: dependence on caregivers for daily care; vulnerability to receiving inadequate care; and potential receipt of care that is misaligned with individual personal wishes or preferences (Brennan et al., 2023; Jia et al., 2021). Importantly, about 85% of the persons living with dementia worldwide do not receive adequate postdiagnostic care and support, which includes advance care planning (ACP; Alzheimer’s Disease International, 2022). Documented barriers to such support include limited availability and accessibility of ACP resources, including the absence of evidence-based guidelines to address the complexity of engaging persons living with dementia in discussions regarding their future care (Palmer et al., 2021; Piers et al., 2018). Although proper incorporation of ACP in the care of people living with dementia provides potential benefits for maintaining quality of life, reducing potential harm, mitigating AD-related symptoms, improving end-of-life outcomes, and minimizing overutilization of healthcare resources, studies have shown that as few as 40%–60% of people living with dementia engage available ACP resources (Dixon et al., 2017; Goldfarb et al., 2019; Lingler et al., 2008; Pettigrew et al., 2020; Sellars et al., 2019).

ACP is a process that allows people to discuss and document their preferences for medical care, based on personal values and life goals (Sudore et al., 2017). A broad conceptualization of ACP encompasses discussions related to legal and medical decisions as well as informal sharing of preferences regarding activities of daily living (ADL) and future ADL-related care needs (Kim et al., 2021). However, uncertainties of the ADRD disease trajectory complicate this process (Anderson et al., 2022). Although ACP does not provide direct assistance regarding interactions with the healthcare system, it supports caregivers to advocate for people living with dementia based on a patient’s personal wishes and carries the potential to mitigate ethical dilemmas associated with proxy decision making (Sellars et al., 2019). Both people living with dementia and their caregivers are generally interested in the discussions related to both legal and informal ACP; however, barriers such as lack of dementia and disease trajectory-related knowledge can prevent people from initiating the process or being receptive to engaging in ACP (Bryant et al., 2023; Dixon et al., 2017; Pettigrew et al., 2020; Phenwan et al., 2020). Moreover, an individual’s personal level of education, as one of the social determinants of health, also influences their likelihood of learning about the disease (Raghupathi & Raghupathi, 2020; Zajacova & Lawrence, 2018).

Sudore et al. (2008) and Schickedanz et al. (2009) have put forth a conceptual framework of ACP based on the transtheoretical behavior change model, which outlines the distinct steps in the process of establishing ACP, including precontemplation, contemplation, preparation, action, and maintenance. Beyond patients themselves, family and friends also play an essential role in ACP, especially at the action stage (Schickedanz et al., 2009; Sudore et al., 2008). Under this model, information needs about health- and care-related choices are identified as potential barriers to engagement in ACP (Schickedanz et al., 2009). In the context of dementia care, two recent systematic reviews highlight that factors influencing caregivers’ behaviors with respect to ACP are not well understood, despite caregivers’ significant role in decision making and care delivery, especially toward advanced disease stages (Gregg et al., 2021; Sellars et al., 2019). Applying the work of Schickedanz and colleagues to ACP in the context of dementia care requires examining factors associated with caregivers’ understanding of care recipients’ planning behaviors and goals of care. This is further underscored by the frequency of co-occurring comorbidities in people with dementia and their need for support from their caregivers in long-term decision making. To that end, a recent synthesis of studies of seriously ill adults with multiple chronic conditions suggests the need to understand how, sociodemographic characteristics as social determinants of health may shape perspectives and discussions about ACP (Murali et al., 2020).

A recent study by Pettigrew et al. (2020) examined factors that influence attitudes toward ACP among caregivers of the people living with dementia and collected data about the caregiver’s disease-related knowledge, education, and perspectives of care preferences in the people living with dementia. The data set from that study provides a unique opportunity for a secondary analysis, informed by the conceptual work of Schickedanz et al. (2009) and Sudore et al. (2008), to assess the impact of dementia health literacy in caregivers of the persons living with dementia on their perceptions of care-related preferences and planning in the people living with dementia. This analysis addresses the following aims: (1) to examine the association between caregivers’ knowledge of dementia and their perceptions about the primary goals for medical care in people living with dementia; (2) to investigate the associations between caregiver sociodemographic characteristics (e.g., years of education and relationship with the persons living with dementia) and their perceptions about the primary goals for medical care in people living with dementia; and (3) to explore the interactions of caregiver’s years of education and their dementia-specific knowledge with caregivers’ perceptions about the primary goals of medical care in people living with dementia.

## Method

### Design

This is a secondary analysis of the data from a cross-sectional descriptive survey study that focused on the people living with dementia and their caregivers’ attitudes toward advanced care planning. This secondary analysis was reviewed and approved by the University of Pittsburgh Institutional Review Board (STUDY24010176).

### Parent Study

Data from the parent study (Pettigrew et al., 2020) were collected from 13 geographically dispersed Alzheimer’s Disease Research Centers (ADRCs) across the United States through surveys with caregivers of people living with dementia. Additional information about those persons living with dementia, such as demographic information and dementia-related clinical characteristics, was provided by each Center using information obtained during standardized, comprehensive assessments collected during the patients’ annual visits to the ADRCs.

A total of 431 caregivers from the ADRCs located in the Southeast, Northeast, Midwest, and West regions of the United States were included. Participants completed the investigator-designed 41-item “Care Planning for Individuals with Dementia” survey containing multiple-choice questions that aimed to examine caregivers’ preferences and perceptions of plans regarding end-of-life care across different stages of dementia and other relevant factors in the people living with dementia, including their knowledge about dementia (Pettigrew et al., 2020). The survey was first developed based on the study team’s clinical and research expertise, and then validated with six caregivers of persons with mild, moderate, or severe dementia and enrolled in the Johns Hopkins ADRC. These six caregivers completed the survey and participated in qualitative interviews about their experiences of completing the survey. Qualitative data were then analyzed for content validation and identified items that require modification to enhance clarity and understandability, and the survey was modified based on caregivers’ feedback.

In the survey, the level of medical care was defined as intensive care (use of all available medical treatments), basic medical care (use of some, such as fluid maintenance, and antibiotics, but not all medical treatments), and comfort care (only use medical treatments to relieve uncomfortable symptoms, such as pain medications). In addition, other variables such as the relationship between caregivers and the persons living with dementia, the levels of impairment of the persons living with dementia (i.e., Clinical Dementia Rating; Morris, 1993), and demographic information of caregivers and the persons living with dementia were collected as part of the Uniform Data Set and utilized as covariates. The survey was administered in person or via phone from September 2015 to September 2017 (Pettigrew et al., 2020).

### Included Data for This Analysis

This secondary analysis focused on the following topics from the parent study survey as predictor variables: care partners’ knowledge and perceptions about ADRD, caregivers’ perception of hospice care, and caregivers’ knowledge about different types of ACP that were completed or not completed for the persons living with dementia. Specifically, care partners’ knowledge about ADRD was assessed with 10 true/false questions and scored based on the correctness of the answer, and care partners’ self-perceived level of ADRD knowledge was assessed categorically (“knowing a lot,” “knowing something,” “knowing very little,” and “knowing nothing”) regarding early, moderate, and severe stages of dementia (Pettigrew et al., 2020). Outcome variables in our analysis were the caregiver’s perceptions of the primary goals of medical care for the persons living with dementia.

### Statistical Analysis

Statistical analysis was conducted with the statistical software SPSS (version 29; IBM Corp, 2023). During the screening of frequency and distribution of the data, categorical data from the survey that contained variability of less than 10% per response choice were combined (Tabachnick & Fidell, 2013). In addition to screening outliers, multiple imputation was utilized for the missing data, specifically including all of the analyzing variables and other auxiliary variables (age, gender, race, and ethnicities of the persons living with dementia) as predicting variables (Madley-Dowd et al., 2019). After the completion of multiple imputation, the generated data were compared with the original data in terms of data distribution, variances, linearity, correlations, and outliers to ensure overall representativeness of the generated data set. The independence, linearity, multicollinearity, and homoscedasticity were evaluated by examining each outcome variable in relationship to the included independent variables, moderators, and covariates, and all assumptions were satisfied during the examination.

Ordinal, binomial, and multinomial logistic regression models were used to examine the relationship of the caregiver’s dementia-related knowledge to their perceptions of the primary goals of medical care of the persons living with dementia and treatment preferences-related discussions that generated those goals. Interaction terms were additionally examined to evaluate whether these relationships were moderated by caregiver’s level of education. Univariate analysis was conducted as preliminary screening, and the outcomes variables that demonstrated *p* ≤ .2 were selected as the candidate variables. Model assessments were conducted with Hosmer–Lemeshow goodness-of-fit test, which determined the final adjusted models based on selected predicting variables.

## Results

### Sample Characteristics

Caregivers (*n* = 431) included in this study had a mean age of 78.3 years, an average of 15.9 years of education, were primarily female (68.7%), and were 88.5% White. In terms of caregivers’ relationships with the persons living with dementia, 74.0% were the spouses of the persons living with dementia, 76.8% lived with the persons living with dementia, and 82.3% maintained daily interactions with the persons living with dementia. Moreover, 95.1% of the caregivers were also the chosen healthcare proxy of those persons living with dementia, with 86.8% of the persons living with dementia having established their medical power of attorney with a legal document and 79.7% of the persons living with dementia having executed a living will. Among the persons living with dementia, 15.1% had no impairment or mild cognitive impairment (MCI) while 36.6% had mild-stage dementia, 29.6% had moderate-stage dementia, and 19.1% had advanced-stage dementia.

The caregiver’s dementia-related knowledge was assessed using both objective measures (10 true/false questions of dementia-related statements) and subjective evaluations (caregiver’s self-reported level of knowledge regarding different stages of dementia). The mean total dementia knowledge score was 8.4 out of 10. Additionally, 57.3% of the caregivers reported that they already knew “a lot about mild-stage dementia,” 49.2% reported that they knew “a lot about moderate-stage dementia,” and 32.9% reported knowing “a lot about advanced-stage dementia.” For hospice-related knowledge, 31.9% of the caregivers said that they knew a lot about it and 79.3% of the caregivers were aware of the bereavement care delivered with hospice services. Lastly, regarding caregivers’ perceptions of the care recipients’ preferences for primary goals of care, 22.7% of the caregivers disclosed that comfort care was preferred at the present time and 81.7% of the caregivers indicated that comfort care would be preferred at the end stage of disease based on the preferences of persons living with dementia. While 50% of the caregivers stated that they were aware of any treatment-related discussions between persons living with dementia and their healthcare providers, 42% of the caregivers noted that further treatment preference-related discussion should be conducted between persons living with dementia and their healthcare providers. Also, 41.3% of the caregivers indicated that further treatment preference-related discussion between them and the persons living with dementia was needed. Additional details about the characteristics of those included variables are presented in Table 1.

**Table 1.** Sample Characteristics of Surveyed Caregivers (*n* = 431)

Variable	Statistic
Caregiver characteristics	
Age (mean, min–max)	78.3, 31–67
Total years of education (mean, min–max)	15.9, 8–23
Sex (%)	
Female	68.7
Male	31.3
Race (%)	
White	88.5
Underrepresented individuals	11.5
Relationship with the persons living with dementia (%)	
Spouse	74.0
Not spouse	26.0
Living arrangement between caregiver and the persons living with dementia (%)	
Together	76.8
Not together	23.2
Frequency of visit/spending time together between caregiver and persons living with dementia (%)	
Daily	82.3
Not daily	17.6
Whether the surveyed caregiver is the chosen healthcare proxy of each patient (%)	
Yes	95.1
No	4.9
Presence of discussion about preferences of persons living with dementia’s medical treatment between person living with dementia and their caregivers (%)	
Yes	78.8
No	21.2
Level of dementia of affiliated patient based on Clinical Dementia Rating^a^ (%)	
No impairment to mild cognitive impairment (scoring: 0, 0.5)	15.1
Mild stage of dementia (scoring: 1)	36.6
Moderate stage of dementia (scoring: 2)	29.6
Severe stage of dementia (scoring: 3)	19.1
Caregiver’s knowledge about dementia	
Total dementia knowledge score (/10)^b^ (mean, min–max)	8.4, 4–6
Self-reported level of knowledge on mild-stage dementia (%)	
Knowing a lot	57.3
Knowing some things, very little, or nothing	42.7
Self-reported level of knowledge on moderate-stage dementia (%)	
Knowing a lot	49.2
Knowing some things, very little, or nothing	50.8
Self-reported level of knowledge on severe-stage dementia (%)	
Knowing a lot	32.9
Knowing some things	45.5
Knowing very little or nothing	21.6
Caregivers’ knowledge about hospice	
Caregivers’ self-reported level of knowledge about hospice care (%)	
Knowing a lot	31.9
Knowing some things	53.2
Knowing very little to nothing	14.9
Caregivers’ awareness of bereavement care and counseling to family and friends with hospice care (%)	
Yes	79.3
No	20.7
Caregiver perceptions of the primary goals of care of the persons living with dementia	
Patients’ preference for level of medical care at present time (%)	
Intensive medical care	31.0
Basic medical care	46.3
Comfort care	22.7
Patients’ preference for level of medical care at end stage of illness (%)	
Not comfort care	18.3
Comfort care	81.7
Whether caregivers think there should be further discussion between caregivers and care recipients about patients’ treatment preferences of not (%)	
Yes	41.3
No	27.7
No, because he/she is no longer capable of having that discussion	31.0
Caregiver’s awareness of discussions between healthcare provider and persons living with dementia about medical treatment preferences (%)	
Yes	30.9
No	50.0
Don’t know	19.1
Whether caregivers think there should be further discussion between persons living with dementia and their healthcare providers about their preferred types of medical treatment or not (%)	
Yes	42.0
No	27.0
No, because he/she is no longer capable of having that discussion	31.0
Advance care planning activities of persons living with dementia	
Establishment of medical power of attorney with legal document (%)	
Yes	86.8
No	13.2
Establishment of living will (%)	
Yes	79.7
No	20.3

*Notes*:

^a^Clinical Dementia Rating is semistructured interview-based assessment that is conducted with both patients and their study partners to assess the severity of dementia in a patient from the perspectives of the patient’s memory, orientation, judgment and problem-solving, community affairs, home and hobbies, and personal care. Each category is rated from 0 (no impairment), 0.5 (very mild impairment), 1 (mild), 2 (moderate), and 3 (severe), and an overall rating is generated in consideration of all categories.

^b^The total dementia knowledge score (10 points possible) is generated based on caregivers’ answers from 10 true/false questions regarding a set of dementia-related statements within the “Care Planning for Individuals with Dementia” survey of the parent study.

### Caregivers’ Perceptions of Medical Treatment Preferences of the Persons Living With Dementia

The results of unadjusted and adjusted models with caregiver’s perceptions about the medical treatment preferences of the persons living with dementia at both the present time and end stage of dementia are presented in Table 2.

**Table 2.** Predictors of the Caregiver’s Perceptions of the Medical Care Preferences of the Person With Dementia Based on Binomial and Ordinal Logistic Regression Models

Predictors or covariates	Caregiver’s perceptions about the medical care preferences of the persons living with dementia at current time (reference group: “comfort care”)		Caregiver’s perceptions about the medical care preferences of the persons living with dementia at end stage of illness (reference group: “comfort care”)	
	Unadjusted	Adjusted^a^	Unadjusted	Adjusted^a^
	OR (95% CI) *p* value	OR (95% CI) *p* value	OR (95% CI) *p* value	OR (95% CI) *p* value
Caregivers’ total dementia knowledge score	1.48 (1.02, 2.12) .06	0.88 (0.75, 1.30) .10	1.38 (1.14, 1.68) .001	1.27 (1.03, 1.55) **.02**
Caregivers’ perceptions of their knowledge about mild-stage dementia				
Knowing a lot	1.26 (0.88, 1.82) .21	—	1.67 (1.00, 2.79) .05	0.79 (0.41, 1.53) .43
Caregivers’ perceptions of their knowledge about moderate-stage dementia				
Knowing a lot	1.48 (1.02, 2.12) .04	2.53 (0.74, 1.62) .48	2.16 (0.93, 5.00) .72	—
Caregivers’ perceptions of their knowledge about severe-stage dementia				
Knowing a lot	1.64 (0.63, 1.68) .91	—	1.37 (0.69, 2.71) .37	—
Knowing some things	1.35 (0.63, 1.68) .91	—	1.53 (0.80, 2.92) .200	—
Caregivers’ perceptions of their knowledge about hospice care				
Knowing a lot	0.77 (0.43, 1.39) .39	—	1.11 (0.50, 2.48) .80	—
Knowing some things	0.76 (0.44, 1.31) .32	—	0.94 (0.43, 2.05) .88	—
Caregivers’ awareness of bereavement care with hospice services				
Yes	1.09 (0.70, 1.68) .71	—	0.58 (0.31, 1.08) .09	0.79 (0.31, 1.53) .43
Caregivers’ years of education	0.88 (0.81, 0.96) .003	0.89 (0.82, 0.97) **.009**	0.99 (0.90, 1.09) .85	—
Caregivers’ sex				
Male	1.17 (0.79, 1.73) .42	—	0.51 (0.30, 0.86) <.001	0.53 (0.31, 0.93) **.03**
Caregiver’s age	1.19 (1.00, 1.03) .03	—	1.00 (1.00, 1.02) .05	0.53 (0.31, 0.93) .31
Caregivers’ relationship with persons living with dementia				
Spouse	0.83 (0.55, 1.23) .35	—	1.32 (0.75, 2.31) .34	—
Caregivers’ living arrangement with persons living with dementia				
Living together	1.35 (0.88, 2.10) .17	—	1.01 (0.56, 1.82) .98	—
Caregivers’ frequency of visits with persons living with dementia				
Daily	0.92 (0.57, 1.49) .74	—	0.85 (0.43, 1.67) .63	—
Level of dementia of				
No impairment and MCI	0.15 (0.08, 0.30) <.001	0.17 (0.09, 0.35) **<.001**	0.91 (0.38, 2.14) .82	—
Mild	0.35 (0.21, 0.60) <.001	0.36 (0.21, 0.62) **<.001**	0.97 (0.48, 2.0) .94	—
Moderate	0.58 (0.34, 1.00) .048	0.58 (0.34, 1.00) .051	1.21 (0.57, 2.57) .62	—
Establishment of power attorney of persons living with dementia				
Yes	0.72 (0.38, 1.39) .32	—	1.21 (0.47, 3.17) .68	—
Establishment of living will of persons living with dementia				
Yes	1.07 (0.63, 1.84) .79	—	1.41 (0.75, 2.75) .28	—
Presence of patients’ treatment preferences between caregiver and persons living with dementia				
Yes	1.57 (0.99, 2.51) .06	1.61 (0.98, 2.64) .06	2.92 (1.50, 5.66) .002	2.59 (1.29, 5.17) **.008**

*Notes*: CI = confidence interval; MCI = mild cognitive impairment; OR = odds ratio; bold values are p-values that are statistically significant in the adjusted models.

^a^Unadjusted variables were excluded either because they had a *p* value greater than .2 or did not enhance the goodness of fit in the adjusted model.

While increased years of education is associated with lower likelihood of caregivers to endorse patients’ preference for intensive or basic care for current treatment (odds ratio [OR] = 0.89, *p* = .009), the same association was not statistically significant for end-stage care preferences. However, caregivers with higher total dementia knowledge scores were 1.27 times more likely (OR = 1.27, *p* = .02) to endorse comfort care as the patients’ end stage preferred medical care. Compared to caregivers of people with severe stage of dementia, caregivers of people with no impairment and MCI were 83% less likely to choose comfort care (OR = 0.17, *p* < .001), and caregivers of people with mild stage of dementia are 63% less likely to choose comfort care (OR = 0.36, *p* < .001). Lastly, if caregivers already had conversations with the persons living with dementia about their treatment preferences, those caregivers were 2.59 times more likely to choose comfort care compared to those caregivers who have not had the conversation (OR = 2.59, *p* = .008). There were no statistically significant interactions between caregivers’ years of education and caregivers’ disease-specific knowledge on their perceptions of medical treatment preferences in persons living with dementia (*p* > .917).

### Caregivers’ Perceptions of Treatment Preference-Related Discussions for the Persons Living With Dementia

The results of both the unadjusted and adjusted models evaluating caregiver’s perceptions of the need for or awareness of the patients’ treatment preference-related discussions are summarized in Table 3. No further interaction effecting was tested because no statistically significant relationship was found between caregivers’ years of education and their perceptions of treatment preference-related discussions.

**Table 3.** Predictors of the Caregiver’s Perceptions Regarding Discussions Related to the Medical Care Preferences of the Person With Dementia With Multinomial Logistic Regression Models

Predictors or covariates	Caregivers’ perceptions of the need for further medical care preference-related discussion between caregivers and persons living with dementia (reference group: “no”)				Caregivers’ awareness of the occurrence of discussions of medical treatment preferences between the persons living with dementia and their healthcare providers (reference group: “no”)				Caregivers’ perceptions of the need of further medical care preference-related discussions between the persons living with dementia and their healthcare providers (reference group: “no”)			
	Unadjusted		Adjusted^1^		Unadjusted		Adjusted^1^		Unadjusted		Adjusted^1^	
	Yes	No, patient unable	Yes	No, patient unable	Yes	Don’t know	Yes	Don’t know	Yes	No, patient unable	Yes	No, patient unable
	OR (95% CI) *p* value	OR (95% CI) *p* value	OR (95% CI) *p* value	OR (95% CI) *p* value	OR (95% CI) *p* value	OR (95% CI) *p* value	OR (95% CI) *p* value	OR (95% CI) *p* value	OR (95% CI) *p* value	OR (95% CI) *p* value	OR (95% CI) *p* value	OR (95% CI) *p* value
Caregivers’ total dementia score	1.04 (0.86, 1.25) .70	1.04 (0.85, 1.26) .72	—	—	0.99 (0.83, 1.17) .88	0.97 (0.79, 1.18) .75	—	—	1.22 (1.01, 1.48) .04	1.12 (0.92, 1.36) .25	1.266 (1.038, 1.544) **.020**	1.117 (0.896, 1.392) .327
Caregivers’ perceptions of their knowledge about mild-stage dementia												
Knowing a lot	0.54 (0.34, 0.88) .01	1.28 (0.75, 2.18) .36	—	—	1.30 (0.84, 2.02) .25	0.60 (0.36, 1.01) .05	0.77 (0.46, 1.30) .33	0.48 (0.27, 0.87) **.016**	0.68 (0.42, 1.11) .13	1.25 (0.74, 2.12) .40	—	—
Caregivers’ perceptions of their knowledge about moderate-stage dementia												
Knowing a lot	0.58 (0.26, 0.94) .03	2.21 (1.31, 3.71) .03	1.27 (0.68, 2.34) .45	2.12 (1.05, 4.27) **.04**	1.23 (0.80, 1.90) .34	0.74 (0.44, 1.24) .26	—	—	0.57 (0.35, 0.93) .02	1.71 (1.02, 2.87) .04	—	—
Caregivers’ perceptions of their knowledge about severe-stage dementia												
Knowing a lot	0.20 (0.10, 0.42) <.001	0.39 (0.21, 0.85) .005	0.17 (0.07, 0.41) **<.001**	0.87 (0.25, 3.05) .82	2.91 (1.51, 5.59) .001	1.08 (0.54, 2.17) .82	2.11 (1.00, 4.46) .051	1.11 (0.50, 2.46) .80	0.25 (0.12, 0.50) <.001	2.89 (1.20, 6.97) .02	0.314 (0.147, 0.668) **.003**	1.765 (0.683, 4.564) .241
Knowing some things	2.94 (0.99, 8.11) .05	1.78 (0.64, 4.98) .27	0.37 (0.18, 0.75) **.006**	1.07 (0.34, 3.31) .91	2.07 (1.10, 3.89) .24	1.10 (0.58, 2.07) .78	1.80 (0.91, 3.54) .09	1.17 (0.59, 2.29) .66	0.46 (0.24, 0.88) .02	1.94 (0.77, 4.36) .17	0.515 (0.256, 1.039) .064	1.412 (0.560, 3.559) .464
Caregivers’ perceptions of their knowledge about hospice care												
Knowing a lot	0.82 (0.35, 1.90) .64	1.28 (0.53, 3.12) .59	—	—	2.96 (1.45, 6.07) .003	2.03 (0.88, 4.67) .10	2.16 (0.99, 4.68) .052	2.17 (0.89, 5.27) .09	1.26 (0.59, 2.67) .55	1.20 (0.55, 2.60) .65	—	—
Knowing some things	0.82 (0.39, 1.72) .59	1.07 (0.47, 2.47) .87	—	—	1.49 (0.75, 2.95) .25	1.38 (0.63, 3.02) .43	1.32 (0.64, 2.71) .45	1.59 (0.70, 3.59) .27	1.43 (0.71, 2.89) .32	1.44 (0.70, 3.00) .32	—	—
Caregivers’ awareness of bereavement care with hospice services												
Yes	1.04 (0.58, 1.86) .89	0.84 (0.45, 1.59) .60	—	—	0.79 (0.45, 1.38) .41	1.25 (0.69, 2.29) .46	—	—	0.63 (0.36, 1.12) .12	0.56 (0.30, 1.05) .07	—	—
Caregivers’ years of education	0.98 (0.89, 1.08) .67	0.98 (0.89, 1.09) .68	—	—	1.02 (0.93, 1.11) .71	1.02 (0.92, 1.13) .71	—	—	1.07 (0.96, 1.18) .22	1.04 (0.94, 1.15) .50	—	—
Caregivers’ sex												
Male	0.57 (0.35, 0.93) .24	0.31 (0.18, 0.55) <.001	0.50 (0.29, 0.88) **.02**	0.34 (0.18, 0.65) **.001**	1.04 (0.66, 1.67) .86	1.11 (0.64, 1.91) .71	—	—	0.91 (0.55, 1.51) .71	0.52 (0.29, 0.92) .03	—	—
Caregiver’s age	0.98 (0.96, 1.00) .04	0.98 (0.96, 1.01) .16	0.96 (0.94, 0.99) **.003**	0.99 (0.97, 1.02) .63	0.98 (0.96, 0.99) .01	0.98 (0.96, 1.00) .12	—	—	1.00 (0.97, 1.01) .25	0.99 (0.97, 1.01) .35	—	—
Caregivers’ relationship with persons living with dementia												
Spouse	1.32 (0.77, 2.26) .32	1.02 (0.58, 1.79) .94	—	—	0.35 (0.21, 0.58) <.001	0.25 (0.14, 0.45) <.001	—	—	1.44 (0.83, 2.49) .20	1.03 (0.59, 1.80) .93	—	—
Caregivers’ living arrangement with persons living with dementia												
Did not live together	0.60 (0.32, 1.12) .12	2.38 (1.34, 4.22) .003	0.42 (0.19, 0.91) **.03**	1.32 (0.64, 2.71) .45	2.72 (1.58, 4.66) <.001	3.64 (2.01, 6.63) <.001	2.71 (1.52, 4.82) **<.001**	3.71 (2.00, 6.89) **<.001**	0.66 (0.36, 1.21) .18	2.11 (1.18, 3.77) 2.11	—	—
Caregivers’ frequency of visits with persons living with dementia												
Daily	1.39 (0.71, 2.75) .34	0.50 (0.27, 0.95) .03	—	—	0.36 (0.20, 0.67) .001	0.24 (0.12, 0.46) <.001	—	—	1.17 (0.59, 2.32) .65	0.49 (0.26, 0.95) .03	—	—
Level of dementia of persons living with dementia												
No impairment and MCI	8.35 (2.27, 30.73) .002	0.02 (0.002, 0.13) <.001	5.24 (1.35, 20.32) **.02**	0.02 (0.002, 0.17) **<.001**	0.71 (0.33, 1.51) .37	0.78 (0.32, 1.90) .59	—	—	9.34 (2.76, 31.55) <.001	0.02 (0.002, 0.13) <.001	7.381 (2.132, 25.553) **.002**	0.019 (0.002, 0.155) **<.001**
Mild	5.42 (1.58, 18.60) .01	0.08 (0.04, 0.20) <.001	3.26 (0.92, 11.54) .07	0.09 (0.04, 0.21) **<.001**	0.75 (0.40, 1.40) .36	0.75 (0.36, 1.59) .45	—	—	6.49 (2.06, 20.49) .001	0.10 (0.05, 0.23) <.001	5.315 (1.655, 17.068) **.005**	0.115 (0.051, 0.256) **<.001**
Moderate	2.43 (0.65, 9.00) .18	0.28 (0.13, 0.57) <.001	1.53 (0.40, 5.89) .54	0.25 (0.12, 0.56) **<.001**	0.75 (0.39, 1.45) .40	0.91 (0.43, 1.94) .81	—	—	3.20 (0.98, 10.39) .05	0.33 (0.16, 0.69) .003	2.789 (0.849, 9.157) .091	0.361 (0.175, 0.746) .06
Establishment of power attorney of persons living with dementia												
Yes	0.66 (0.33, 1.33) .25	1.14 (0.49, 2.64) .77	—	—	1.56 (0.79, 3.09) .20	1.44 (0.54, 3.79) .46	—	—	0.62 (0.28, 1.34) .22	1.00 (0.42, 2.39) 1.00	—	—
Establishment of living will of persons living with dementia												
Yes	0.97 (0.54, 1.73) .92	1.23 (0.65, 2.32) .52	—	—	1.03 (0.60, 1.77) .92	1.16 (0.60, 2.24) .65	—	—	1.27 (0.69, 2.33) .45	1.32 (0.71, 2.47) .38	—	—
Presence of patients’ treatment preferences between caregiver and care recipients												
Yes	0.40 (0.21, 0.74) .003	0.73 (0.36, 1.45) .37	0.39 (0.19, 0.78) **.01**	0.67 (0.28, 1.57) .36	4.91 (2.41, 9.97) <.001	1.26 (0.69, 2.28) .45	4.84 (2.31, 10.13) **<.001**	1.49 (0.79, 2.82) .22	0.52 (0.27, 1.00) .05	0.96 (0.48, 1.86) .87	0.519 (0.248, 1.085) .080	0.930 (0.439, 1.972) .850

*Notes*: CI = confidence interval; MCI = mild cognitive impairment; OR = odds ratio; bold values are p-values that are statistically significant in the adjusted models.

^a^Unadjusted variables were excluded either because they had a *p* value greater than .2 or did not enhance the goodness of fit in the adjusted model.

#### Further treatment preference-related discussions between the persons living with dementia and their caregivers

Caregivers who identified as knowing a lot about severe stage of dementia were 83% less likely (OR = 0.17, *p* < .001) and caregivers who stated that they knew some things about severe stage of dementia were 62.8% less likely (OR = 0.37, *p* = .006) to endorse the need for further discussions with the persons living with dementia compared to those caregivers who knew very little to nothing. Caregivers who said they knew a lot about moderate stage of dementia were 2.12 times more likely (OR = 2.12, *p* = .036) to indicate no further discussions were needed because persons living with dementia were no longer capable of having such discussions. Caregivers who did not live with the persons living with dementia were 58% less likely (OR = 0.42, *p* = .03) to endorse the need further discussions, and caregivers who already had discussions with the persons living with dementia about their medical treatments when they no longer have decisional capacity were 61% less likely to endorse the need further discussions (OR = 0.39, *p* = .01). Caregivers of patients who had no impairment or MCI were 5.24 times more likely (OR = 5.24, *p* = .02) to indicate a need for further discussions compared to caregivers of persons living with dementia at the severe stage.

#### Caregivers’ awareness of discussions of medical treatment preferences between persons living with dementia and their healthcare providers

Caregivers with a high level of knowledge about mild-stage dementia were 52% less likely (OR = 0.48, *p* = .02) to indicate “don’t know” about it compared to those caregivers with little or no knowledge. While caregivers who did not live with the persons living with dementia were 2.71 times more likely to know about such discussions (OR = 2.71, *p* < .001) compared to caregivers who lived with the persons living with dementia, those caregivers who did not live with the persons living with dementia were also 3.71 times more likely to indicate “don’t know” (OR = 3.71, *p* < .001) about this discussion compared to caregivers who lived with persons living with dementia. Lastly, caregivers who had discussions with the persons living with dementia about their treatment preferences for future care when the persons living with dementia is incapacitated were 4.84 times more likely (OR = 4.84, *p* < .001) to know about discussions of medical care preferences between the persons living with dementia and their healthcare providers, compared to those caregivers who did not have discussions with the persons living with dementia.

#### Further medical care preference-related discussions between the persons living with dementia and their healthcare providers

Caregivers with higher dementia knowledge scores are 1.27 times more likely (OR = 1.27, *p* = .02) to be aware of previous treatment preference-related discussions between the persons living with dementia and their healthcare clinicians. Caregivers with a higher level of knowledgeabout severe-stage dementia were 69% less likely (OR = 0.31, *p* = .003) to indicate the need for additional discussions between persons living with dementia and their healthcare providers, compared to caregivers with little or no knowledge about advanced-stage dementia. Caregivers of the persons living with dementia who had no impairment or MCI were 7.38 times more likely (OR = 7.38, *p* = .002) and caregivers of the persons living with dementia who had mild stage of dementia were 5.32 times more likely (OR = 5.32, *p* = .01) to indicate the need for further medical care preference-related discussions between the persons living with dementia and their healthcare providers compared to caregivers of the persons living with advanced-stage disease. Caregivers of the persons living with dementia who are at earlier stages were also less likely to endorse that there was no further need for discussion when a patient becomes incapacitated compared to caregivers of patients with advanced-stage disease (no impairment and MCI: OR = 0.02, *p* < .001; mild: OR = 0.12, *p* < .001; moderate: OR = 0.36, *p* = .01).

## Discussion

This study examined associations between factors such as dementia-care knowledge, caregivers’ perceptions about treatment-related discussions, and medical care preferences in persons living with dementia. Overall, the results indicate that caregivers with higher knowledge about dementia, or dementia health literacy, were more likely to engage in ACP to clarify the preferences of the persons living with dementia, and to indicate preferences for comfort care. Moreover, caregivers with less disease-specific knowledge and those who were involved with patients at earlier stages of disease indicated that additional medical care preferences-related discussions were needed. Lastly, while caregivers who live with care recipients may not always be aware of patients’ treatment preferences, or related discussions between patients and their healthcare clinicians, they are more likely to seek this information if they have previously talked about these topics with their care recipients.

While caregivers maintain an essential role in advocating for people living with dementia regarding goal-concordant levels of medical care when the patient is no longer capable, previous literature indicates that health literacy, including disease-specific knowledge, influences the engagement of people living with dementia in ACP and promotion of suitable medical care based on people’s personal values (Fried et al., 2021; Sudore et al., 2006; Tetrault et al., 2022). Our study enriches this understanding by illustrating that caregivers with more dementia-related knowledge are likely to indicate comfort care as the preference of care recipients during end stage, and endorse that further treatment preference-related discussions are needed with healthcare clinicians. Discussions related to patients’ treatment-related preferences are necessary when preventing the delivery of medical care that does not align with an individual’s personal values or ensure comfort, and involving caregivers during those conversations ensures long-term continuity of care in people living with dementia (Hickman et al, 2023; Wright et al., 2008). Furthermore, with the announcement of the Guiding an Improved Dementia Experience Model by the Centers for Medicare & Medicaid Services, incorporating disease-specific education as a component of standardized dementia care will hopefully improve caregivers’ understanding of dementia-specific knowledge and further foster discussions to promote person-centered care plans (Centers for Medicare & Medicaid Services, 2023). Such efforts should also acknowledge that caregivers’ knowledge may accumulate over time due to repeated experiences in the course of caregiving.

Moreover, these data suggest that caregivers’ perceptions of medical treatment-related discussions and preferences of people living with dementia change across stages of disease. Specifically, caregivers of patients with earlier stages of dementia were more likely to identify curative or disease-directed care as a current preference, while those caregivers were also likely to indicate the need for further relevant discussions. In addition, caregivers were more likely to identify comfort care for end-stage dementia if they already had treatment preference-related discussions with their care recipients. This aligns with the conceptual work of Schickedanz and colleagues (2009) and is consistent with the literature about the dynamic, continuous process of ACP-related discussions and the requirement for routine updates of treatment-related goals among healthcare professionals, family members, and caregivers (Kilackey et al., 2020). Moreover, timely discussions also facilitate adequate end-of-life care for people living with dementia that promotes comfort and dignity while eases some of the emotional burdens of the family members and caregivers (Bamford et al., 2018, Zhang et al., 2024). Moving forward, application of Shickendanz’s framework in the context of dementia should give additional consideration to the influence of ADRD-related cognitive decline on the ability of people living with dementia to reflect their personal choices for both formal and informal care (Schickedanz et al., 2009).

We additionally found that caregivers of people living with earlier stages of dementia indicate the need for further treatment-related discussions between patients and clinicians. This aligns with the previous qualitative study by Tetrault et al. (2022), suggesting that it is important to discuss decisions related to future care during the early stages of the disease, and that timing is crucial given the progressive loss of the patient’s cognitive and functional abilities. Although the process of figuring out and planning for dementia care is complicated, the progression of B-amyloid neuroimaging and the potential for blood biomarker testing in the near future for disease diagnosis provide increasing opportunities for patients and their families to contemplate and discuss their specific care preferences as they progress along the ADRD trajectory (Grill & Lingler, 2024; Olsson et al., 2016). Supporting the connective relationship among caregivers, patients, and healthcare providers may further facilitate this ACP process (Schickedanz et al., 2009).

Importantly, healthcare providers also play a necessary role in cultivating discussions with people living with dementia about their treatment-related preferences and encouraging the early initiation of ACP-related discussions among persons living with dementia and their families. While our study noted the need for additional treatment preference-related discussions from caregivers’ perspectives, a previous descriptive study also found the need for physicians to be more knowledgeable and proactive regarding their approaches to ACP in people with mild to moderate stages of dementia (Cavalieri et al., 2002). While the 2016 Centers for Medicare and Medicaid policy change introducing billing for ACP offers the opportunity for providers to promote ACP discussions, the prevalence of those ACP discussions remains low (Palmer et al., 2021). However, effective ACP communications also require appropriate guidelines and training oriented to clinicians when implementing standardized care. Our findings suggest it is important to consider clients’ demographic characteristics and personal background, like the living arrangement between the caregivers and care receipts, when initiating treatment preference-related discussions to truly address the needs of each individual patient and their families in an efficient manner (Pettigrew et al., 2020). Existing ACP programs remain out of reach for many and do not adequately meet the needs of people with various backgrounds, especially those from minoritized communities (Moss et al., 2021; Palmer et al., 2021). Given that dementia education and ACP-related practices may vary by backgrounds and personal experiences, future studies should explore caregivers’ perspectives across different ages, sexes, genders, races, ethnicities, and income levels to ensure effective implementation of ACP interventions across populations.

Lastly, especially for living arrangements, while cohabitants have often been assumed to have more opportunities to discuss patients’ care-related preferences or participate in conversations with their caregivers, our results suggested the opposite. One potential explanation could be the living arrangement of the persons living with dementia being home versus an institution. ACP-related discussions and documentation are encountered or triggered more frequently with changes in the care setting or level of care, which could potentially lead to caregivers’ greater awareness of those ACP-related conversations (van Soest-Poortvliet et al., 2015). Although additional information was not collected within this analyzed data set, future exploration on the nature and quality of the relationship between people living with dementia and their caregivers, and their impact on caregivers’ awareness of ACP-related conversations and care choices (Vernon et al., 2019). Since ACP for dementia requires participation and decision-making from both people living with dementia and their caregivers, future considerations on the dyadic relationship could identify the barriers and facilitators of implementing ACP in this population (Fried et al., 2021; Liu et al., 2024).

### Limitations

This study has limitations. Although caregivers of people living with various disease stages were included in this analysis, the data set was a cross-sectional survey; it will be important to study the evolution of changes in care decisions over time through longitudinal assessment with consideration of caregivers’ baseline disease-specific knowledge. Moreover, it was necessary for some variables to be statistically transformed or combined categorically to ensure the match of assumptions prior to conducting the main effect analysis. However, this could also lead to a potential loss of contextual information, diminish the richness of the data, and impede a comprehensive understanding of research questions. Also, because data set was not originally designed for interaction testing, statistical power or variance of each variable may have limited our ability to detect significant interactions. We were also unable to control for factors such as prior exposure to dementia education programs and duration of time providing care, both of which may influence dementia health literacy. Lastly, the surveyed sample was mainly White and highly educated. While this is reflective of an overarching pattern of overrepresentation of non-Latinx White adults in ADRCs (Arce Rentería et al., 2023), future studies should strive for greater inclusivity of persons from minoritized communities to ensure more representative findings that could effectively guide ACP-related interventions and policy developments.

## Conclusion

While engaging in ACP is an essential process to promote medical care that aligns with personal values and preferences of people living with dementia, it is also necessary to understand caregivers’ perspectives about ACP-related components and incorporate them into the process of dementia care. In this analysis, the importance of caregivers’ dementia-specific knowledge was a particular focus, and the findings demonstrate the need for involving caregivers in disease-specific education after their loved ones’ disease diagnosis. Additionally, the study results demonstrate the critical importance of engaging in these treatment preference-related discussions relatively early in the disease course and including their families, as well as assistance from healthcare providers.

## Data Availability

The data used in this study are available upon request through communications with the principal investigators of the parent study (Pettigrew et al., 2020). The analytic method of this study is available to share upon contact of the corresponding author of this manuscript. This study was not preregistered.
